# Transcriptomic and metabolomic analyses reveal the differential accumulation of phenylpropanoids and terpenoids in hemp autotetraploid and its diploid progenitor

**DOI:** 10.1186/s12870-023-04630-z

**Published:** 2023-12-05

**Authors:** Qing Tang, Ying Xu, Feng Gao, Ying Xu, Chaohua Cheng, Canhui Deng, Jiquan Chen, Xiaoge Yuan, Xiaoyu Zhang, Jianguang Su

**Affiliations:** 1 Institute of Bast Fiber Crops, Chinese Academy of Agricultural Sciences, Changsha, 410205 Hunan China; 2 Center for Industrial Hemp Science and Technology Innovation, Institute of Bast Fiber Crops, Chinese Academy of Agricultural Sciences, Changsha, 410205 Hunan China; 3 Yunnan Academy of Industrial Hemp, Kunming, 650214 Yunnan China

**Keywords:** *Cannabis sativa*, Transcriptome, Metabolomics, Autopolyploidization, Phenylpropanoid, Terpenoid

## Abstract

**Background:**

*Cannabis sativa*, a dioecious plant that has been cultivated worldwide for thousands of years, is known for its secondary metabolites, especially cannabinoids, which possess several medicinal effects. In this study, we investigated the autopolyploidization effects on the biosynthesis and accumulation of these metabolites, transcriptomic and metabolomic analyses were performed to explore the gene expression and metabolic variations in industrial hemp autotetraploids and their diploid progenitors.

**Results:**

Through these analyses, we obtained 1,663 differentially expressed metabolites and 1,103 differentially expressed genes. Integrative analysis revealed that phenylpropanoid and terpenoid biosynthesis were regulated by polyploidization. No substantial differences were found in the cannabidiol or tetrahydrocannabinol content between tetraploids and diploids. Following polyploidization, some transcription factors, including nine bHLH and eight MYB transcription factors, affected the metabolic biosynthesis as regulators. Additionally, several pivotal catalytic genes, such as *flavonol synthase*/*flavanone 3*-*hydroxylase*, related to the phenylpropanoid metabolic pathway, were identified as being modulated by polyploidization.

**Conclusions:**

This study enhances the overall understanding of the impact of autopolyploidization in *C. sativa* and the findings may encourage the application of polyploid breeding for increasing the content of important secondary metabolites in industrial hemp.

**Supplementary Information:**

The online version contains supplementary material available at 10.1186/s12870-023-04630-z.

## Background

Cannabis (*Cannabis sativa* L.), belonging to the family Cannabaceae, is one of the oldest domesticated food and cash crops [1]. Presently, cannabis is utilized not only for producing high-grade paper, textiles, edible oil, and protein powder but also for the production of drugs, health products, cosmetics, and food additives because of its beneficial cannabinoids. Cannabinoids are a class of secondary metabolites in cannabis, and more than 150 cannabinoids have been identified to date [2, 3]. Cannabidiol (CBD) is one of the most important cannabinoids and has recently attracted considerable attention owing to its positive effects in the treatment of epilepsy, depression, joint pain, and other diseases [4]. Notably, medicinal cannabinoid preparations, including Marinol®, Satiex®, and Epidiolex®, have been developed for treating anorexia, multiple sclerosis, and pediatric seizure disorders, respectively [5, 6]. Another large group of secondary metabolites in cannabis is terpenes, which are not only responsible for the scent and flavor of cannabis products but also possess therapeutic properties [7]. Flavonoids, phenol family members, are also an important group of compounds in cannabis that show promising therapeutic effects [8]. These secondary metabolites are mainly produced and accumulated in female cannabis flowers [9].

Polyploidy refers to individuals with three or more complete sets of chromosomes (one from each parent or ancestor) in their somatic and germline cells, and exists in vascular plants [10, 11]. Polyploidization is an important mechanism for speciation, genome evolution, and biodiversity maintenance, and it is also a classic strategy for the de novo domestication of crops [12, 13]. There are two main mechanisms of polyploidization, one of which is the failure of cells to divide after chromosome replication, resulting in the doubling of chromosomes in the cell, thus forming an autopolyploid. The other mechanism involves the hybridization of different species, resulting in allopolyploids [14]. In contrast to diploid plants, polyploid plants generally exhibit more vigorous growth, larger fruit, higher yields, and greater environmental adaptability [15, 16]. In addition to morphological changes, polyploidy also induces changes in the content of secondary metabolites. Therefore, breeders often induce polyploidy to generate medicinal or agricultural crop varieties with larger organs and improved secondary metabolite profiles [17–19].

Cannabis is a dioecious annual plant that is open wind-pollinated. There are 10 pairs of chromosomes in cannabis cells, consisting of nine autosomes and one pair of sex chromosomes (XX or XY) [20, 21]. Historically, conventional breeding methods for hemp have included mass selection, inbreeding, hybrid breeding, cross breeding, and marker-assisted breeding [22]. The cultivation of new cannabis varieties often requires considerable time and effort. To overcome these challenges, breeders have adopted chromosome doubling technology to facilitate the development of new polyploid cannabis strains [11, 23]. Chromosome doubling technology has been shown to influence the morphology of cannabis considerably, affecting the size of inflorescences and leaves and the composition of secondary metabolites. However, data on the effects of polyploidization on the content of metabolites, such as tetrahydrocannabinol (THC) and CBD, are inconsistent. Although studies have reported that polyploidization increases the cannabinoid ratio [23], conflicting results have shown no change in the THC content [11, 24].

Recently, integrative transcriptomic and metabolomic analysis has facilitated the identification of key genes and metabolic pathways related to the biosynthesis of secondary metabolites in plants [25, 26]. Past studies in the field of polyploidy have mainly focused on elucidating the relationship between polyploidization and gene expression using transcriptomic techniques [27–29]. However, combined metabolomic and transcriptomic analysis has been used to elucidate gene expression and metabolic differences in polyploids [30, 31]. Although preliminary studies have shown that polyploidization can induce phenotypic and metabolic changes in cannabis plants, the mechanisms of these changes have yet to be elucidated.

Current methods for comparing differences in gene expression levels across the transcriptome rely on detecting statistically significant differences in RNAseq read abundances at each locus. To quantify expression levels, normalization methods, such as dividing read abundance by concentration (e.g., FPKM or basemean), are necessary. Following normalization, the gene expression data indicate the transcript’s abundance relative to the total transcriptome. This concentration-based normalization approach is widely applied to detect differences in gene expression induced by gene copy number and has been extensively used to elucidate mechanisms of polyploidization-induced changes in numerous plant species [30–33]. In this study, the concentration-based normalization approach was employed to investigate the gene expression, and integrative transcriptomic and metabolomic analysis was used to elucidate the mechanisms of polyploidization-induced changes in cannabis plants, including the effect on polyploidy-responsive key functional genes and regulators of metabolic pathways. Our research provides a theoretical and practical basis for developing new cannabis varieties with a high content of important secondary metabolites through the use of polyploid breeding techniques.

## Results

### Polyploidization affects the content of cannabinoids

**Fig. 1:**
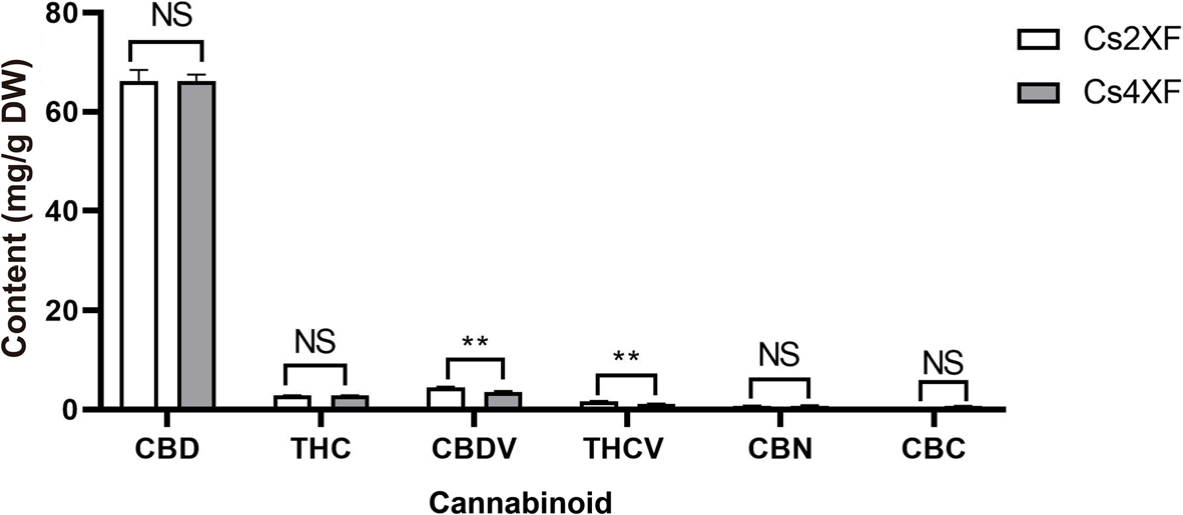
Cannabinoid content of tetraploids and diploids. Data are presented as means ± standard error (*n* = 9). Statistical significance is calculated using Student’s t-test; ‘**’ and ‘NS’ indicate a significant difference at the *p* < 0.01 level and no significant difference at the *p* < 0.05 level, respectively. DW, dry weight

In the context of this study, the impact of polyploidization on cannabinoid content was examined. No statistically relevant differences in the CBD and THC content were detected between tetraploids and diploids (Fig. 1, Table S1). Specifically, the CBD content of the tetraploid and diploid plants was 66.17 ± 1.31 and 66.25 ± 2.20 mg g^−1^, respectively, and the average THC content was approximately 2.8 mg g^−1^. The cannabichromin (CBC) and cannabiol (CBN) contents of the plants were about 0.6 and 0.8 mg g-1, respectively, and were not significantly affected by polyploidization. The cannabidivarin (CBDV) and tetrahydrocannbivarin (THCV) content of diploids were 4.5 ± 0.1 and 1.7 ± 0.1 mg g-1, respectively. Notably, 22.2 and 35.3% decreases were observed in CBDV and THCV content of tetraploids, respectively, compared with that of diploids. Overall, these results show that polyploidization had a limited influence on cannabinoid biosynthesis.

### Metabolomic alterations in industrial hemp following autopolyploidization

**Fig. 2:**
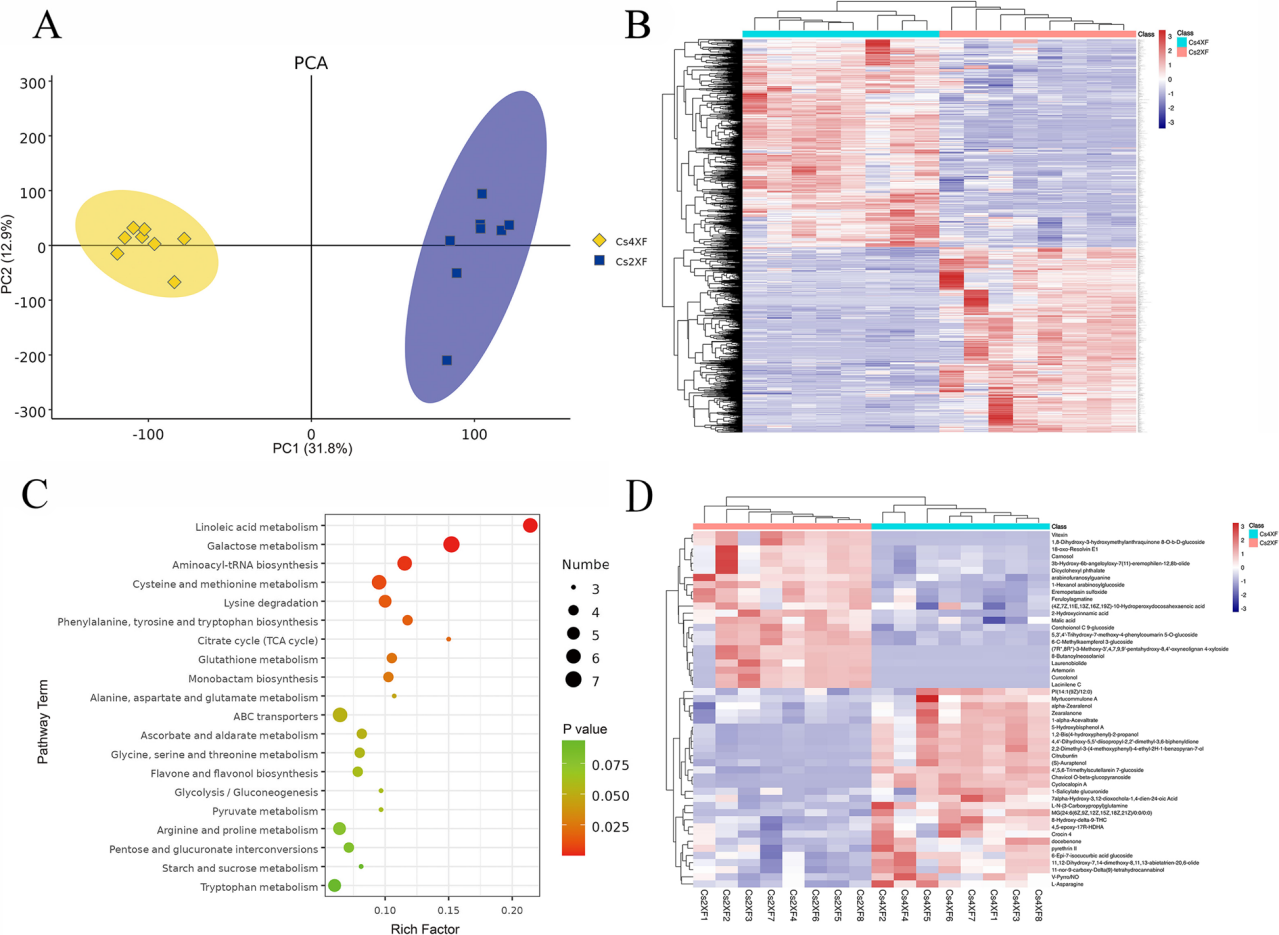
Differentially expressed metabolites (DEMs) following polyploidization. **A** Principal component analysis (PCA) score plot of metabolite profiles between Cs4XF and Cs2XF. **B** Heat map of DEMs based on hierarchical clustering analysis. **C** Kyoto Encyclopedia of Genes and Genomes (KEGG) pathway annotation of DEMs. Copyright permission has been granted for related KEGG images. **D** Heat map of top 50 DEMs based on hierarchical clustering analysis

By means of liquid chromatography–mass spectrometry (LC–MS), the effect of polyploidization on secondary metabolites in the industrial hemp variety DMG265 was evaluated. Principal component analysis (PCA) showed a clear clustering between female tetraploid (Cs4XF) and diploid (Cs2XF) samples, with the first (PC1) and second (PC2) principal components accounting for 31.8 and 12.9% of the total variation, respectively (Fig. 2A). In the inflorescences, 1,663 differentially expressed metabolites (DEMs) were identified between Cs4XF and Cs2XF (Fig. 2B). Pathway analysis demonstrated that 108 DEMs were annotated in Kyoto Encyclopedia of Genes and Genomes (KEGG) database (http://www.kegg.jp/) pathways, including those of linoleic acid metabolism, galactose metabolism, and flavone and flavonol biosynthesis (Fig. 2C). The top 50 DEMs (28 upregulated and 22 downregulated) between Cs4XF and Cs2XF, according to the variable importance in projection (VIP) value, were chosen for further analysis. Based on these 50 metabolites, we were able to effectively distinguish between the two groups (Fig. 2D). Among the fifty DEMs, four terpene lactones, three cinnamic acids and derivatives, three flavonoids, three sesquiterpenoids, one diterpenoid, and one lignin glycoside were identified (Table S2). These DEMs were mainly enriched in pathways related to the biosynthesis of terpenes, phenylpropanoids, and flavonoids. It was worth noting that several of these DEMs were possess particular medicinal properties such as myrtucommulone A and crocin-4.

### Differentially expressed genes related to polyploidization in industrial hemp

**Fig. 3:**
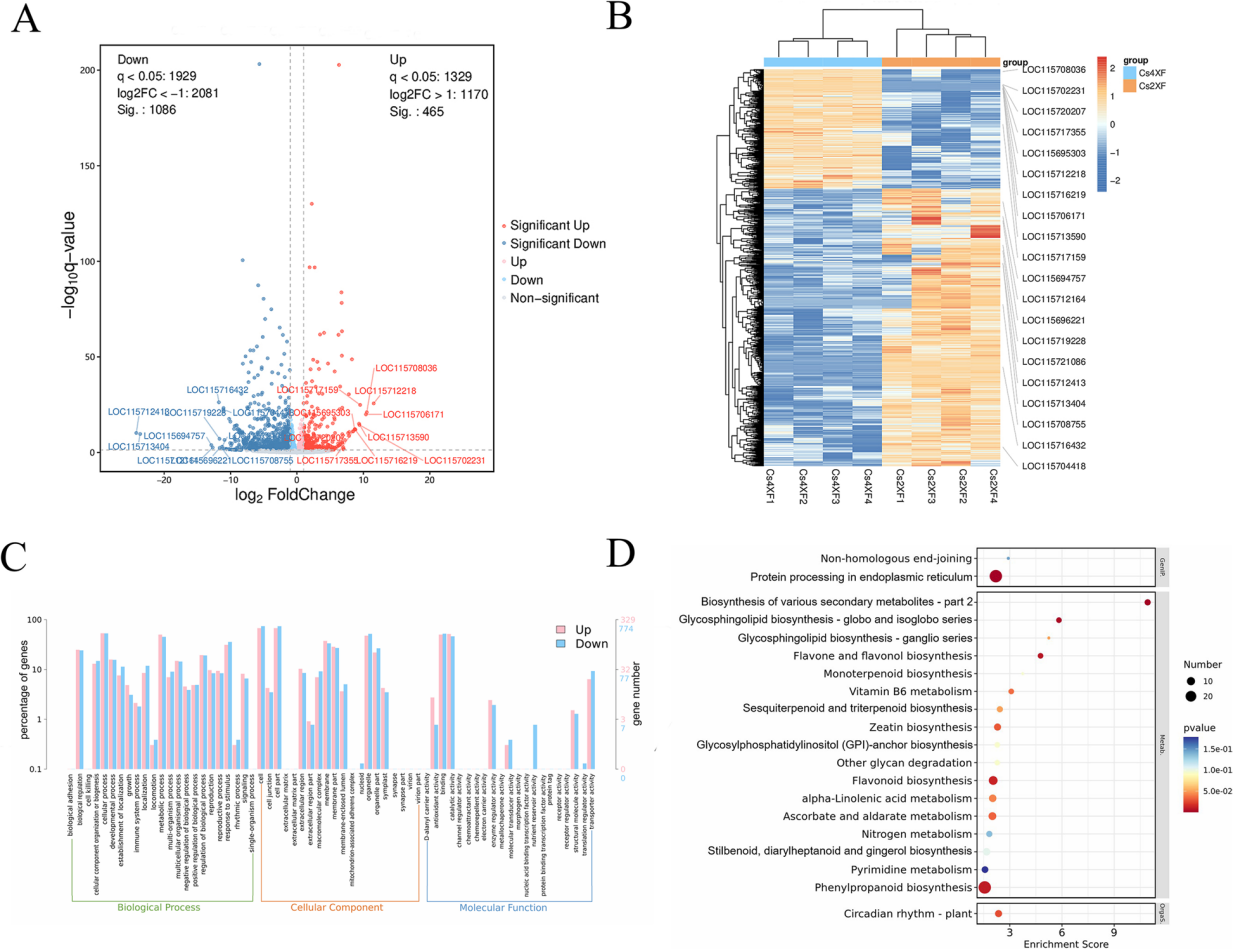
Differentially expressed genes (DEGs) following polyploidization. **A** Volcano map of DEGs. **B** Heatmap of DEGs based on hierarchical clustering analysis. **C** Gene ontology (GO) classification of DEGs. **D** Kyoto Encyclopedia of Genes and Genomes (KEGG) pathway analysis of DEGs. Copyright permission has been granted for related KEGG images

High-throughput transcriptome sequencing was performed to shed further light on the effect of polyploidization on gene expression and the biosynthesis of secondary metabolites. A total of 52.77 G clean reads with a Q30 percentage > 92% were obtained after quality screening of the raw data. Based on these data, 19,205 genes were identified and quantified, among which 1,551 differentially expressed genes (DEGs) (465 upregulated and 1,086 downregulated) (Table S3) were detected between Cs4XF and Cs2XF (Fig. 3A, B).

Gene ontology (GO) (http://geneontology.org/) enrichment analysis was utilized to define the functions of the DEGs (Fig. 3C), and 1,103 DEGs were successfully annotated, including 329 upregulated and 774 downregulated genes. Specifically, the DEGs were primarily enriched in cellular process, metabolic process, and response to stimulus in the “Biological Process” category. Furthermore, KEGG pathway analysis demonstrated that the polyploidy-responsive genes were abundant in the flavone and flavonol, flavonoid, phenylpropanoid, sesquiterpenoid and triterpenoid, and monoterpenoid biosynthesis pathways (Fig. 3D).

### Integrated metabolomic and transcriptomic analysis of phenylpropanoid, flavonoid, and terpenoid biosynthesis

**Fig. 4:**
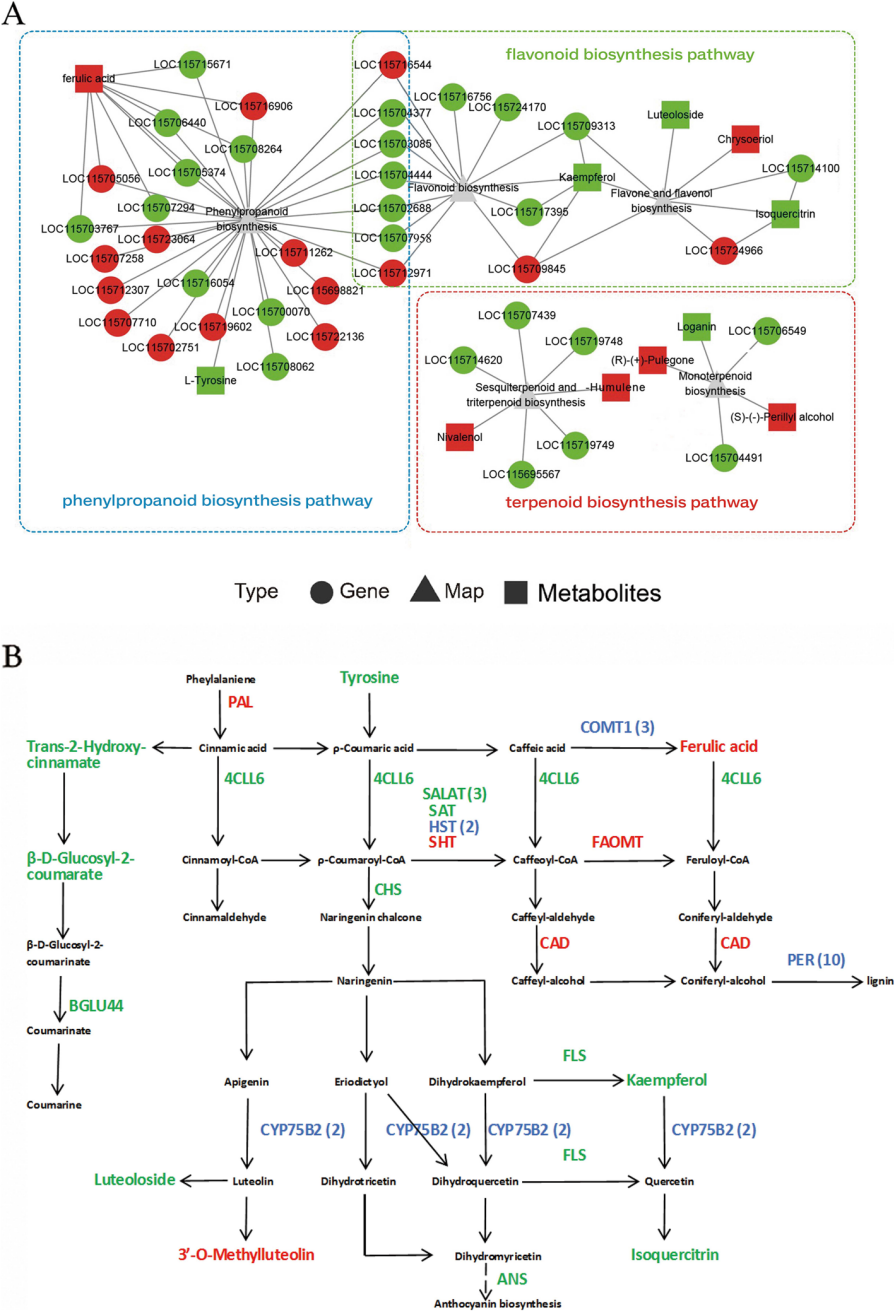
Integrative metabolomic and transcriptomic analysis of phenylpropanoid, flavonoid, and terpenoid biosynthesis pathways. **A** The results of correlation analysis between differentially expressed genes (DEGs) and differentially expressed metabolites (DEMs) (*p* ≤ 0.05). The red dots and squares represent upregulated DEGs and DEMs, respectively, while the green dots and squares represent downregulated DEGs and DEMs, respectively. **B** Genes and metabolites in the phenylpropanoid biosynthesis pathway were modulated by polyploidization. The red and green words indicate upregulated and downregulated genes or metabolites, respectively. A mix of upregulation and downregulation for the gene is represented in blue, and values in parentheses represent the gene number

Integrative transcriptomic and metabolomic analysis was performed to identify polyploidy-responsive metabolic pathways and genes. Several key metabolic pathways, including the cannabinoid, phenylpropanoid, flavonoid, and terpenoid biosynthesis pathways, had attracted considerable attention. In the present study, a few metabolites and several synthetic or catalytic genes in these metabolic pathways were substantially affected by polyploidization (Fig. 4A).

In the phenylpropanoid and flavonoid biosynthesis pathways, 8 DEMs and 29 DEGs were markedly altered (Fig. 4B). Ferulic acid, kaempferol, trans-2-hydroxy-cinnamate, 3′-O-methylluteolin, β-D-glucosyl-2-coumarate, and isoquercitrin were markedly altered after polyploidization. Additionally, several pivotal catalytic genes (*PAL*, *4CL*, *CHS*, *CYP75B2*, *CAD*, *FAOMT*, and *PER*) were considerably affected by polyploidization (Fig. 4B, Table S4). In the monoterpenoid, sesquiterpenoid, and triterpenoid biosynthesis pathways, five DEMs, namely, (R)-( +)-pulegone, (S)-(-)-perillyl alcohol, loganin, nivalenol, and α-humulene, and seven DEGs were influenced by polyploidization (Fig. 4A, Table S5). Overall, these results indicate that biosynthesis of phenylpropanoid, flavonoid, and terpenoid was influenced by polyploidization.

**Fig. 5:**
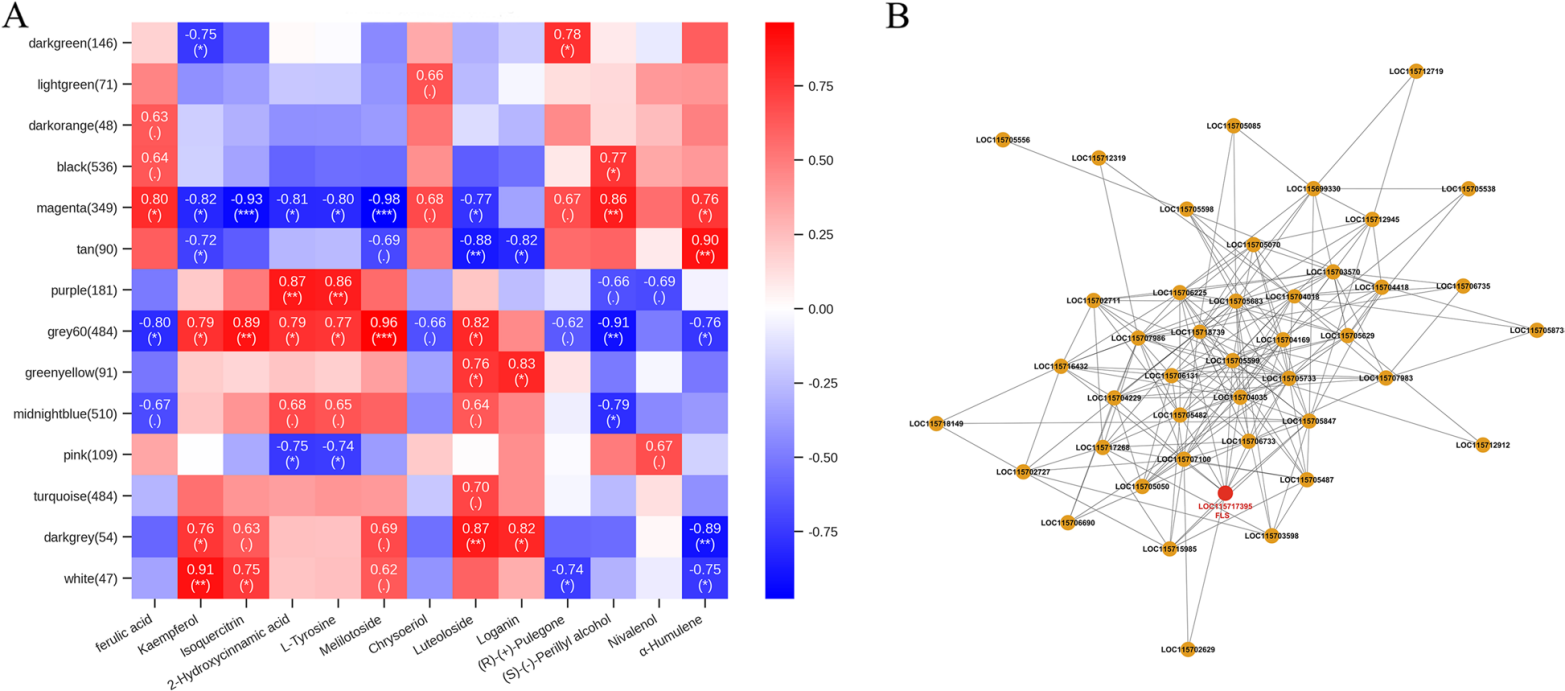
Weighted gene co-expression network analysis (WGCNA). **A** Identification of modules associated with 13 differentially expressed metabolites (DEMs) in the phenylpropanoid, flavonoid, and terpenoid biosynthesis pathways. Each cell contains the corresponding correlation and *p*-value. ‘*’, ‘**’, and ‘***’ indicate significant differences at the *p* < 0.05, 0.01, and 0.001 levels, respectively. **B** Visualization of the network among the most connected genes (core genes) in the Gray60 module

To identify genes with high topological-overlap similarity in polyploidization, a weighted gene co-expression network analysis (WGCNA) was carried out, integrating the transcriptomic and metabolomic data in a co-expression network for all genes. Fourteen modules of co-expressed genes (Table S6) with distinct expression patterns were identified. The gene significance (GS) score was used to evaluate the correlation between the expression of genes and metabolites. Based on this correlation, the tendency of accumulation of the most positively correlated metabolite(s) with the genes of the modules was shown in Fig. S1. The genes in the Gray60 module showed the most significant positive correlation with the 13 DEMs in the phenylpropanoid, flavonoid, and terpenoid biosynthesis pathways (Fig. 5A). Within this group, the *flavonol synthase*/*flavanone 3*-*hydroxylase* (*FLS*) gene was one of the most highly connected genes (Fig. 5B). This indicated that *FLS* had a influence on the 13 DEMs in this group. In addition, GO and KEGG analyses were employed for the functional annotation of the Gray60 module genes (Fig. S2). The results of GO analysis revealed that these genes were primarily enriched in the sesquiterpene biosynthetic process and sesquiterpene synthase activity categories. Furthermore, KEGG analysis showed that the Gray60 module genes were primarily enriched in the biosynthesis of various secondary metabolites-part 2 and monoterpenoid biosynthesis pathways. The results suggest that the Gray60 module had a strong correlation with terpenoid biosynthesis.

### Transcription factors associated with polyploidization in industrial hemp

In the present study, 59 polyploidy-altered transcription factors (TFs) were identified, including 21 upregulated and 38 downregulated TFs (Fig. S3A). Similar to the DEGs, the number of downregulated TFs was higher than that of the upregulated TFs, among which bHLH (one upregulated and eight downregulated) and MYB (one upregulated and seven downregulated) were the most abundant TFs.

As molecular switches, TFs play a crucial role in modulating plant secondary metabolism [34]. Therefore, a network of TFs and metabolites (|PCC|> 0.917) was generated to determine the effects of TFs on polyploidy-responsive metabolites, and the statistical significance of the correlations is shown in Table S7. The network comprised 45 TFs and 143 DEMs, of which VRN1 (B3 family), PTL (trihelix family), and PRE6 (bHLH family) were highly connected to the DEMs (Fig. S3B). These TFs likely contribute to changes in secondary metabolites, such as terpenoids, phenylpropanoids, and flavonoids.

### Polymerase chain reaction analysis

The levels of expression of the selected genes were verified using quantitative real-time polymerase chain reaction (qRT-PCR) (Fig. S4). The qRT-PCR and transcriptome data showed a high correlation, supporting the reliability of our RNA-seq analysis results. Moreover, the results suggest that the selected genes were useful for studying terpenoid biosynthesis, phenylpropanoids, and flavonoids in tetraploids and diploids.

## Discussion

### Polyploidization did not increase the content of cannabinoids in industrial hemp

Cannabinoids are a unique class of secondary metabolites in cannabis. THC and CBD are the two major cannabinoids and generally produced from their corresponding acids, THCA and CBDA, through nonenzymatic decarboxylation, promoted by light and heat. THCA and CBDA are synthesized from cannabigerolic acid (CBGA) by tetrahydrocannabinolic acid synthase (THCAS) and cannabidiolic acid synthase (CBDAS), respectively [35]. Notably, *THCAS* evolves from the ancestor gene *CBDAS*, and they have a high degree of similarity at the nucleotide and amino acid levels [36, 37]. In the genome, *THCAS* and *CBDAS* are closely related and located in the retrotransposon enrichment region [38, 39]. Multiple *THCAS* and *CBDAS* gene loci are detected in the cannabis genome, and large sequence variations are observed among *CBDAS* gene copies [39–41]. Thus, the synthesis efficiency of the two cannabinoids is determined by the characteristics of their respective enzymes after translation of the allelic gene.

In this study, the metabolomic and transcriptional analyses demonstrated that polyploidization had a limited effect on cannabinoid synthesis. Although past studies have shown that polyploidy can increase CBD content or reduce THC content [11, 23, 42], several recent findings coincide with our results and indicate that it is not the multiple copies of the genes, but rather the functional forms of the THCAS and CBDAS proteins that play a key role in the synthesis of tetrahydrocannabinolic acid (THCA) and cannabidiolic acid (CBDA), respectively [41, 43]. These findings support the hypothesis that there is a co-dominant allele for each synthetase that is the predominant cause of THCA and CBDA synthesis. Thus, selecting functional alleles may be more effective than chromosome doubling in increasing cannabinoid content. However, polyploidization increased the area and thickness of the leaves and increased the size and deepened the color of the inflorescences (Fig. S5). These results show that, although polyploidization did not affect the synthesis of cannabinoids in industrial hemp, it might increase the inflorescence and leaf yield.

### Autopolyploidization substantially and intricately altered phenylpropanoid biosynthesis 

In addition to cannabinoids, cannabis contains several metabolites of pharmacological interest, including terpenoids and phenylpropanoids [44, 45]. The phenylpropanoid biosynthesis pathway produces several metabolites, including coumarins, lignin, and flavonoids [46–48]. In the present study, polyploidization was shown to affect the expression patterns of genes and metabolites related to the phenylpropanoid and flavonoid pathways (Fig. 4B).

Specifically, a decrease was observed in the content of trans-2-hydroxy-cinnamate, β-D-glucosyl-2-coumaratein, and the two precursors for the synthesis of coumarin, in tetraploids. Additionally, the expression of the coumarin biosynthesis gene encoding BGLU44 was downregulated, indicating a potential decrease in coumarin content after polyploidization. Furthermore, autopolyploidization increased the expression modes of critical genes related to lignan biosynthesis, including *FAOMT*, *CAD*, and nine *PER* genes (Table S4). Moreover, compared with diploids, autotetraploids had higher levels of ferulic acid, the precursor of sinapyl and coniferyl alcohols. This suggests a higher lignan content in the autopolyploids than in the diploids. A higher lignan content is also observed in *Isatidis radix*, a common herb used in traditional Chinese medicine, post polyploidization [31].

Although flavonoid biosynthesis in cannabis is not well established, a general pathway has been proposed [8, 44]. We observed a drop in the levels of some metabolites in autopolyploids, such as kaempferol, isoquercitrin, and luteoloside, but an increase in others, including 3′-O-methylluteolin. Similarly, polyploidization significantly affected the expression of key catalytic genes associated with flavonoid biosynthesis, including *FLS*, *PAL*, *4CLL6*, *CHS*, and *CYP75B2*. Metabolomic analysis identified 34 flavonoids (14 upregulated and 20 downregulated) among the DEMs (Table S8), indicating that the biosynthesis of flavonoids in cannabis was considerably affected by polyploidization. Specifically, the *FLS* gene was highlighted through WGCNA. In flavonoid biosynthesis, the formation of flavonols and anthocyanins is catalyzed by FLS and dihydroflavonol 4-reductase [49]. The decreased expression of the *FLS* gene was the main reason for the low kaempferol and isoquercitrin content in the Cs4XF progeny.

Previous studies have mainly reported the accumulation of phenylpropanoids in several plants, following polyploidization [30, 31, 50–52]. However, our results show that polyploidization had a complicated influence on the phenylpropanoid biosynthesis pathway in industrial hemp. This complexity was closely related to polyploidy-responsive TFs (Fig. S3). In this study, nine bHLH, eight MYB, three WRKY, and two ERF TFs were identified. Notably, bHLH80 (CsbHLH83) was correlated with kaempferol and isoquercitrin, and ZAT18 (C2H2 family) was correlated with ferulic acid (Fig. S3B). These results indicate that bHLH80 and ZAT18 were involved in the phenylpropanoid biosynthesis pathway. Similarly, several studies have revealed that these TF families are related to the phenylpropanoid biosynthesis pathway [34, 53, 54]. However, the roles of the differently expressed TFs require further study.

### Autopolyploidization significantly altered terpenoid biosynthesis in industrial hemp

Terpenoids are important aromatic compounds that account for the aroma and flavor of cannabis, with well over 120 terpenoids already identified in the plant [2, 7]. The pharmacological effects of the terpenoids in cannabis and their synergistic effects with cannabinoids are currently being evaluated [55]. As a hot topic in pharmacology, there is an increased demand for medical products containing cannabis-derived compounds with desirable pharmacological properties. Terpenoids are the huge group of isoprenoids compounds, which built from isoprene as structural units and modified by glycosylation, hydroxylation, acylation, dehydrogenation and so on. Monoterpenoids and sesquiterpenoids are volatile only when not conjugated to polar or large (> 200 Da) moieties, on the other hand, higher order terpenoids generally lack volatile [56]. While GC–MS analysis is primarily limited to thermally stable non-polar volatiles, such as fatty acid methyl esters, essential oils and similar samples, LC–MS proves more valuable and suitable for the analyses of non-volatile compounds like phenylpropanoid and flavonoid. Numerous papers and application notes have demonstrated the applicability of LC–MS in studying terpenoids [54, 57–59]. In our study, we used the LC–MS data to analyze the terpenoids and elucidate how polyploidization influences their biosynthesis.

In the polyploidy offspring, significant fluctuation was detected in the terpenoid content, as well as in gene expression in the relative pathways. We found that five DEMs (including α-humulene) and seven DEGs (including *TPS7*) were enriched in the monoterpenoid (ko00902) and sesquiterpenoid-triterpenoid (ko00909) biosynthesis pathways (Fig. 4A). Further analysis of the metabolome data revealed that polyploidization caused an significantly change in many terpenoid compounds, including 15 monoterpenoids, 44 sesquiterpenoids, and 15 triterpenoids (Table S8). Similarly, polyploidization has been shown to cause considerable variation in the terpenoid content of several other plants [60–62].

## Conclusions

In this study, integrative transcriptomic and metabolomic analysis was used to elucidate the mechanisms of polyploidization-induced changes in industrial hemp plants, including 1,663 DEMs and 1,103 DEGs in the metabolic pathways of secondary metabolites. The transcriptomic and metabolomic analyses indicated that, although terpenoid and phenylpropanoid biosynthesis were considerably affected, polyploidization had a limited effect on cannabinoid biosynthesis. Furthermore, TFs may form a dynamic regulatory network that controls the gene expression required for the biosynthesis of metabolites. Overall, the results of this study contribute to a greater understanding of the impact of autopolyploidization in industrial hemp. However, polyploidization-induced changes such as DNA methylation, small interfering RNAs, and microRNAs were not characterized in this study. Additional research that includes epigenetic effects and post-transcriptional controls is necessary to further elucidate the mechanism of polyploidization-induced changes in cannabis. With the deepening of research, ploidy breeding technology will likely play an important role in the breeding of industrial hemp for some desirable characteristics such as terpene manipulation and biomass improvements.

## Methods

### Plant materials and cultivation

This study took place in a laboratory setting, with the aim of determining the mechanisms of polyploidization-induced changes in cannabis plants. The industrial hemp variety DMG265 (2n = 2x = 20) used in this study was donated by the National Germplasm Bank of the Institute of Bast Fiber Crops, Chinese Academy of Agriculture Sciences. DMG265 is a dioecious CBD-rich variety and is commonly used as a diploid donor. The autotetraploid hemp material was induced using colchicine as described previously [23]. Briefly, the seeds of DMG265 were planted in pots and incubated under indoor white fluorescent lighting (light duration, 18 h; average light intensity, 100 µmol m^−2^s^−1^; temperature, 24 ± 2 °C). After the emergence of two true leaves, the apical growing points of the seedlings were treated with 0.2% (w/v, pH 6.0) colchicine solution every 6 h for 24 h. After 50 d, the young leaves at the top of the plant were subjected to flow cytometric analysis to detect the DNA content and ploidy. The ploidy of the tetraploids of different genotypes and their clones was assessed three times, and the results showed that their ploidy was stable (Fig. S5B).

Healthy diploid (Cs2x) and tetraploid (Cs4x) female plants were selected as mother plants for clonal propagation. Twenty cuttings from the mother plant in the vegetative stage were placed in a Jiffy seeding block for rooting and covered with a transparent plastic dome to maintain high humidity. The cuttings were fully rooted approximately 3 weeks later. In the fifth week, eight vigorous and uniform clones of diploid and tetraploid genotypes were transferred into pots. Plants were maintained in the vegetative growth phase (light duration, 18 h; average light intensity, 200 µmol m^−2^s^−1^) for 4 weeks and then switched to reproductive growth (light duration, 12 h; average light intensity, 450 µmol m^−2^s^−1^). After nine weeks, the female inflorescences of each clone were separately sampled and frozen in liquid nitrogen for transcriptome and metabolome analyses. The seedlings were planted in a warm (24 ± 2 °C), ventilated room under a constant and uniform supply of water and nutrients.

### Cannabinoid extraction and content analysis

The standards of cannabinoids (purity ≥ 99.5%) were purchased from Cerilliant Corporation (Texas, USA). Standard stock solutions of these compounds were prepared at a concentration of 1.0 mg/mL in methanol and stored in at -20 °C. These solutions were diluted in methanol to 10 ug/mL working solutions prior to instrumental analysis. All other common chemicals and solvents were of HPLC grade and obtained from Sinopharm Chemical Reagent Co., Ltd (Shanghai, China).

Three inflorescences (approximately 15 cm) from each plant were mixed as a biological replicate (eight replicates per genotype) and dried in the shade for three days, followed by the removal of stems and debris. Then the samples were dried to constant mass (± 2%) in an air convection oven at 60 ± 1 °C for at least 1 h. Thereafter, the samples were ground, sieved through a < 0.25 mm filter. 200 mg of milled sample was heated at 150 ± 1 °C for ten minutes, and extracted in a glass tube using 4 mL of methanol after cooling, then ultrasonicated at 30 °C for 10 min. After standing for 1 h, the mixture was centrifuged at 2920 × g for 5 min, and the supernatant was collected and diluted to 10 mL with methanol. The diluted supernatant was filtered with a 0.45 µM filter and analyzed using high-performance liquid chromatography (HPLC).

This analysis was conducted using an Agilent modular model 1260 system equipped with a chromatographic column (Zorbax sb-c18, 250 mm × 4.6 mm, 5 μm) and a photo diode array (PDA) detector. The column was kept at 25 °C, and the isocratic elution was adopted with the mobile phase of acetonitrile-0.1% acetic acid in water (75:25) at flow rate of 0.8 mL/min. The injection volume was 10 µL. More specifically, the detection wavelength was set at 220 nm. The typical chromatograms of several cannabinoids, including CBD and THC, were provided in Fig. S6.

### Metabolomic analysis of Cannabis sativa 

HPLC-grade methanol, formic acid, water and acetonitrile were purchased from Thermo Fisher Scientific Company (Waltham, MA, USA), and L-2-chlorophenylalanine was purchased from Shanghai Hengchuang Biotechnology Co., Ltd (Shanghai, China). All other chemicals and solvents were analytically pure or chromatographically grade.

A 20 μL of internal standard (L-2-chlorophenylalanine, 0.06 mg/mL; methanol configuration) was added to 80 mg sample with 1 mL methanol/aqueous solution (7:3, V/V) in a 1.5 mL eppendorf tube. The mixture was pre-cooled at -20 °C for 2 min and then ground with two small steel balls at 60 Hz for 2 min, ultrasonicated in ice-water bath for 10 min, and placed at -20 °C for 120 min. After centrifuging at 4 °C (13000 rpm) for 10 min, 150 μL of supernatant was collected using crystal syringes, filtered through a 0.22 μm organic phase pinhole filter and transferred to LC injection vials. The vials were stored at -80 °C until LC–MS analysis. QC samples were prepared by mixing aliquot of the all samples to be a pooled sample.

The metabolome analysis was completed by Shanghai Lu-Ming Biotech Co., Ltd. (Shanghai, China). An AB ExionLC (AB Sciex, MA, USA) coupled with Q-Exactive Plus mass spectrometer equipped with heated electrospray ionization (ESI) source (Thermo Fisher Scientific, Waltham, MA, USA) was used to analyze the metabolic profiles in both ESI positive and ESI negative ion modes. An ACQUITY UPLC HSS T3 column (100 mm × 2.1mm, 1.8 μm) was employed in both positive and negative modes. The binary gradient elution system was composed of (A) water (containing 0.1% formic acid, V/V) and (B) acetonitrile (containing 0.1% formic acid, V/V), and separation was achieved using the following gradient: 0.01 min, 5% B; 2 min, 5% B; 4 min, 30% B; 8 min, 50% B; 10 min, 80% B; 14 min, 100% B; 15 min, 100% B; 15.1 min, 5% and 16 min, 5% B. The flow rate was 0.35 mL/min and the column temperature was 45 ℃. The injection volume was 2 μL. The mass range was from m/z 100 to 1,200. The resolution was set at 70,000 for the full MS scans and 17,500 for HCD MS/MS scans. The Collision energy was set at 10, 20 and 40 eV. The mass spectrometer operated as follows: spray voltage, 3800 V ( +) and 3000 V ( −); sheath gas flow rate, 35 arbitrary units; auxiliary gas flow rate, 8 arbitrary units; capillary temperature, 320 °C; Aux gas heater temperature, 350 °C; S-lens RF level, 50.

The original LC–MS data were processed by software Progenesis QI V2.3 (Nonlinear, Dynamics, Newcastle, UK). Main parameters of 5 ppm precursor tolerance, 10 ppm product tolerance, and 5% product ion threshold were applied. Compound identification was based on precise mass-to-charge ratio (m/z), isotopic distribution, and secondary fragments using the human metabolome database (HMDB), Lipidmaps (V2.3), Metlin, and self-built databases. The extracted data were then further processed by removing any peaks with a missing value (ion intensity = 0) in more than 50% in groups, by replacing zero value by half of the minimum value, and by screening according to the qualitative results of the compounds. Compounds with resulting scores below 36 (out of 60) points were also deemed to be inaccurate and removed. A data matrix was combined from the positive and negative ion data.

The matrix was imported in R (v 3.2.0) to carry out principle component analysis (PCA) to depict the overall distribution among the samples and the stability of the whole analysis process. Orthogonal partial least-squares-discriminant analysis (OPLS-DA) and partial least-squares-discriminant analysis (PLS-DA) were conducted to distinguish the metabolites that differ between groups. To prevent overfitting, 7-fold cross-validation and 200 response permutation testing (RPT) were used to evaluate the quality of the model. Variable importance of projection (VIP) values obtained from the OPLS-DA model were utilized to rank the overall contribution of each variable to group discrimination. A two-tailed Student’s T-test was further used to verify whether the metabolites of difference between groups were significant. Differential metabolites were selected with VIP > 1.0 and *p* < 0.05. Pathway enrichment analysis of the DEMs was carried out using the KEGG database, and metabolic pathways with *p* < 0.05 were considered significantly enriched.

### RNA isolation, sequencing, and differentially expressed gene analysis

Four different clones per genotype were sampled from four biological replicates. Total RNA extraction was performed using TRIzol reagent (Invitrogen, USA), following the manufacturer’s instructions. Libraries were constructed using the VAHTS Universal V5 RNA-seq Library Prep kit for Illumina (Vazyme, China), with reference to the manufacturer’s protocol. Additionally, cDNA library construction and transcriptome sequencing were performed by OE Biotech Co., Ltd. (Shanghai, China). The libraries were sequenced on an Illumina Novaseq 6000 platform, and 150 bp paired-end reads were collected. Approximately 6 Gb of raw bases were generated for each sample; however, more than 40 M clean reads were retained per sample for subsequent analysis after quality screening. Using HISAT2 [63], the clean reads were successfully mapped to the cs10 reference genome (https://ftp.ncbi.nlm.nih.gov/genomes/all/GCF/900/626/175/GCF_900626175.2_cs10/GCF_900626175.2_cs10_genomic.fna.gz). The fragments per kilobase of transcript per million mapped reads of each gene were calculated, and the read counts of each gene were concluded via HTSeq-count [64].

The package DESeq2 V1.22.2 [65] was employed to calculate basemean, foldchange, *p*-value, and Q value, and genes with Q < 0.05 and foldchange > 2 or foldchange < 0.5 were identified as prominent DEGs. Hierarchical cluster analysis of the DEGs was applied to examine the expression patterns of genes across groups and samples using R software (v 3.2.0). Furthermore, GO enrichment and KEGG pathway enrichment analysis of the DEGs were performed using R (v 3.2.0) based on the hypergeometric distribution. Subsequently, GO terms and KEGG pathways with an adjusted *p*-value ≤ 0.05 were recognized as significantly enriched, and R (v 3.2.0) was applied for drawing the column diagram and bubble diagram of the significant enrichment terms and pathways.

### Integrated metabolomic and transcriptomic analysis

Based on the transcriptome and metabolome data, Pearson’s correlation analysis was conducted to confirm the connection between DEGs and DEMs. A correlation network was built using Cytoscape (The Cytoscape Consortium, USA, version 3.8.0).

The WGCNA R package was also implemented to construct a co-expression network. The Pearson’s correlations between the gene modules and the phenotype data (the DEMs) were computed, and significantly correlated modules were identified based on a threshold of |correlation coefficient|> 0.3 and *p* < 0.05.

### Quantitative real-time PCR analysis

For verification of the expression patterns of the DEGs, 14 genes, including 2 transcriptional factors and 11 genes related to terpene and phenylpropanoid biosynthesis, were selected for qRT-PCR analysis, and a TUB gene was selected to be an internal control [66]. All primer sequences are tabulated in Table S9. The qRT-PCR analysis was performed via the application of the iTaq™ Universal SYBR® Green Supermix (Bio-Rad, USA) on an iq5 multicolor real-time PCR system (Bio-Rad). The 2^− ΔΔCT^ method was implemented to calculate the relative expression levels of each gene. The analysis was repeated three times for each sample.

### Statistical analysis

Data were analyzed using SPSS Statistics 26.0 (SPSS Inc., Chicago, IL) and presented as means ± standard error of at least three measurements. Statistical significance was calculated using Student’s t-tests, and statistical differences were considered significant if *p* < 0.05.

### Supplementary Information

**Additional file 1: Supplementary Figure 1.** Scatterplot of gene significance score versus module membership. **Supplementary Figure 2.** GO and KEGG enrichment of genes in the grey60 module. **Supplementary Figure 3.** Analysis of transcription factors (TFs) associated with polyploidization. **Supplementary Figure 4.** Quantitative real-time polymerase chain reaction (qRT-PCR) validation of selected genes. **Supplementary Figure 5.** Morphological characteristics of *Cannabis sativa* autotetraploid and its diploid progenitor. **Supplementary Figure 6.** The typical chromatogram of several cannabinoids. **Supplementary Table 1.** Cannabinoids content (mean ± SE) for dried flower material of *Cannabis sativa* diploids and atutotetraploids analyzed in duplicated (*n*=9) by HPLC. **Supplementary Table 4.** DEGs related to phenylpropanoid biosynthesis. **Supplementary Table 5.** DEGs related to monoterpenoid, and sesquiterpenoid and triterpenoid biosynthesis pathways. **Supplementary Table 9.** The primers of genes for qRT-PCR.

**Additional file 2: Supplementary Table S2.** Top 50 differential expressed metabolites (DEMs).

**Additional file 3: Supplementary Table S3.** Differential expressed genes (DEGs).

**Additional file 4: Supplementary Table S6.** Genes of module.

**Additional file 5: Supplementary Table S7.** Correlation of polyploidy-altered TFs and metabolites (|PCC |> 0.917).

**Additional file 6: Supplementary Table S8.** Polyploidization-modulated terpenoids and flavonoids.

## Data Availability

All LC–MS/MS raw data are available in OMIX (https://ngdc.cncb.ac.cn/omix/) with accession number PRJCA018055 (https://ngdc.cncb.ac.cn/omix/preview/SrNd5OSJ). The RNA-seq dataset was deposited in the NCBI Sequence Read Archive (SRA, http://www.ncbi.nlm.nih.gov/Traces/sra) with accession number PRJNA872904 (https://www.ncbi.nlm.nih.gov/bioproject/PRJNA872904).

## Abbreviations

CBC: Cannabichromin
CBD: Cannabidiol
CBDA: Cannabidiolic acid
CBDAS: Cannabidiolic acid synthase
CBDV: Cannabidivarin
CBN: Cannabiol
DEG: Differentially expressed gene
DEM: Differentially expressed metabolite
*FLS*: *Flavonol synthase*/*flavanone 3*-*hydroxylase*
GO: Gene ontology
GS: Gene significance
HPLC: High-performance liquid chromatography
KEGG: Kyoto Encyclopedia of Genes and Genomes
LC–MS: Liquid chromatography–mass spectrometry
OPLS-DA: Orthogonal partial least-squares-discriminant analysis
PCA: Principal component analysis
PDA: Photo diode array
qRT-PCR: Quantitative real-time PCR
RNA-seq: RNA sequencing
RPT: Response permutation testing
TF: Transcription factor
THC: Tetrahydrocannabinol
THCA: Tetrahydrocannabinolic acid
THCAS: Tetrahydrocannabinolic acid synthase
THCV: Tetrahydrocannbivarin
WGCNA: Weighted gene co-expression network analysis
VIP: Variable importance in projection
