# Supplementary material for: Transcriptomic and metabolomic analyses reveal the differential accumulation of phenylpropanoids and terpenoids in hemp autotetraploid and its diploid progenitor
Source: BMC Plant Biol. 2023 Dec 5;23:616. doi: 10.1186/s12870-023-04630-z (PMC10696708; doi:10.1186/s12870-023-04630-z)
Supplement: Supplementary file 1 — Additional file 1: Supplementary Figure 1. Scatterplot of gene significance score versus module membership. Supplementary Figure 2. GO and KEGG enrichment of genes in the grey60 module. Supplementary Figure 3. Analysis of transcription factors (TFs) associated with polyploidization. Supplementary Figure 4. Quantitative real-time polymerase chain reaction (qRT-PCR) validation of selected genes. Supplementary Figure 5. Morphological characteristics of Cannabis sativa autotetraploid and its diploid progenitor. Supplementary Figure 6. The typical chromatogram of several cannabinoids. Supplementary Table 1. Cannabinoids content (mean ± SE) for dried flower material of Cannabis sativa diploids and atutotetraploids analyzed in duplicated (n=9) by HPLC. Supplementary Table 4. DEGs related to phenylpropanoid biosynthesis. Supplementary Table 5. DEGs related to monoterpenoid, and sesquiterpenoid and triterpenoid biosynthesis pathways. Supplementary Table 9. The primers of genes for qRT-PCR. [file 12870_2023_4630_MOESM1_ESM.docx]

Supplementary Material

Transcriptomic and metabolomic analyses reveal the differential accumulation of phenylpropanoids and terpenoids in hemp autotetraploid and its diploid progenitor

Qing Tang^1,2^, Ying Xu^1^, Feng Gao^3^, Ying Xu^1^(YGX), Chaohua Cheng^1,2^, Canhui Deng^1^, Jiquan Chen^1^, Xiaoge Yuan^1^, Xiaoyu Zhang^1^, Jianguang Su^1,2*^

^1^ Institute of Bast Fiber Crops, Chinese Academy of Agricultural Sciences, Changsha 410205, Hunan, China

^2^ Center for Industrial Hemp Science and Technology Innovation, Institute of Bast Fiber Crops, Chinese Academy of Agricultural Sciences, Changsha 410205, Hunan, China

^3^ Yunnan Academy of Industrial Hemp, Kunming 650214, Yunnan, China

*** Correspondence:** Jianguang Su: [jgsu2016@163.com](mailto:jgsu2016@163.com)


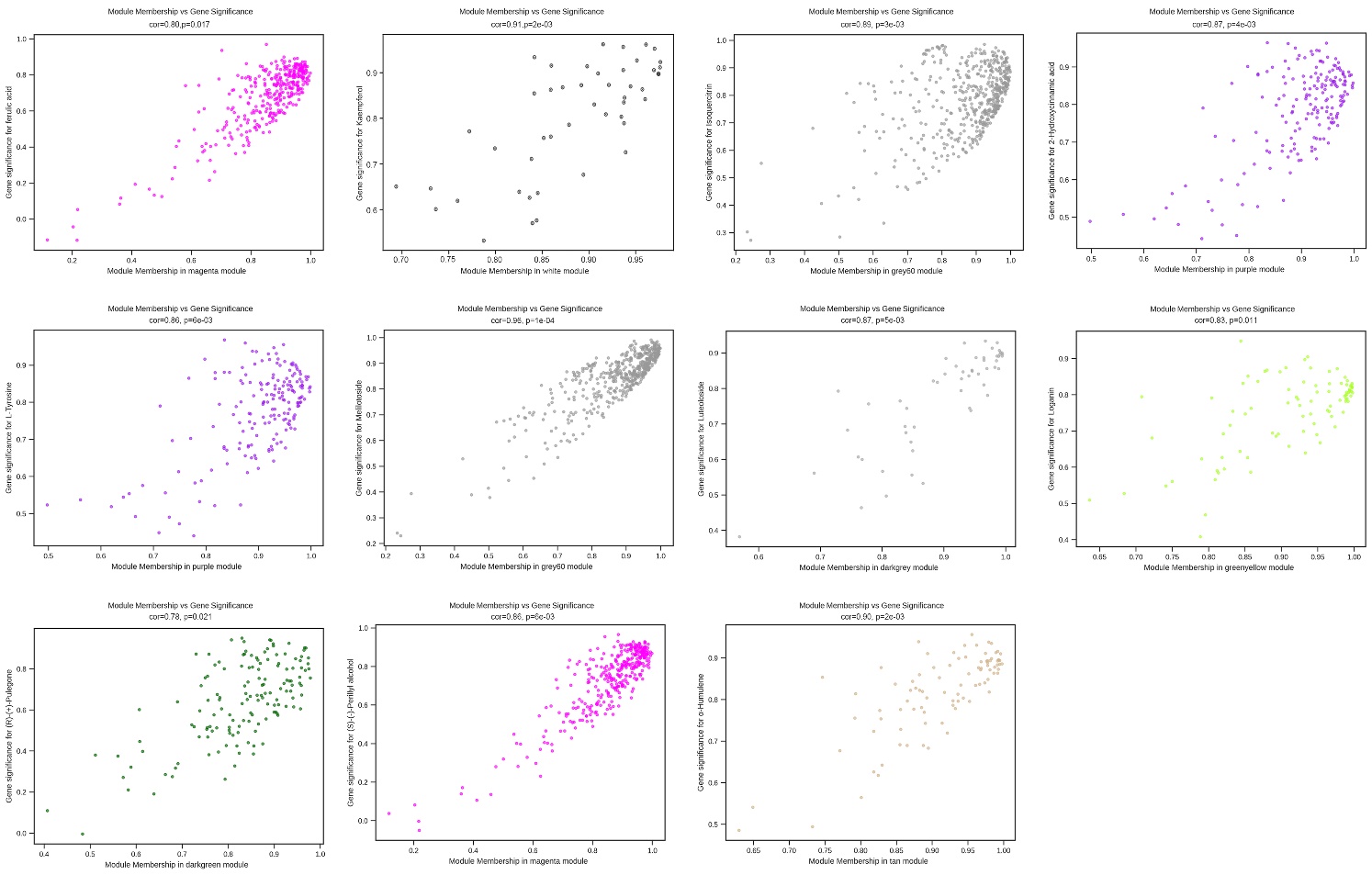


**Supplementary Figure 1.** Scatterplot of gene significance score versus module membership.


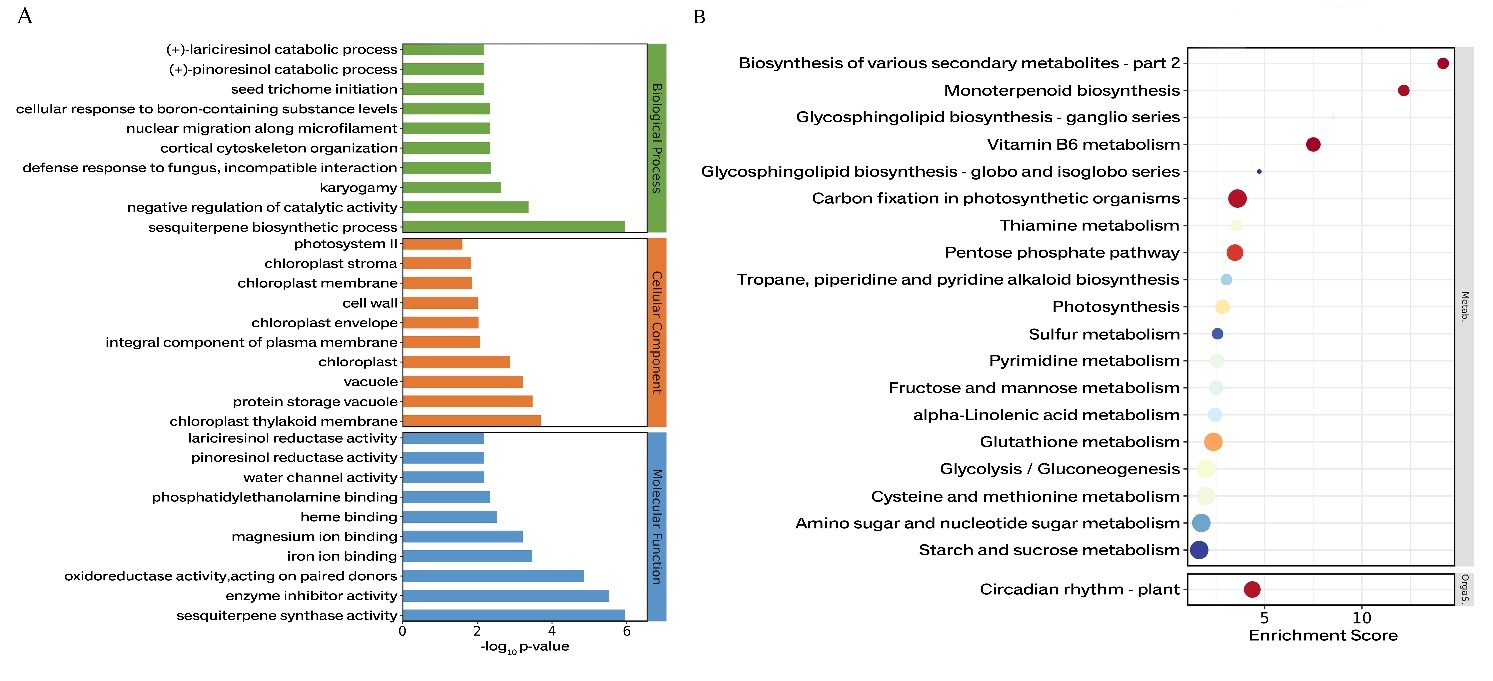


**Supplementary Figure 2.** GO and KEGG enrichment of genes in the grey60 module. **(A)** GO enrichment. **(B)** KEGG pathway. Copyright permission has been granted for related KEGG images.


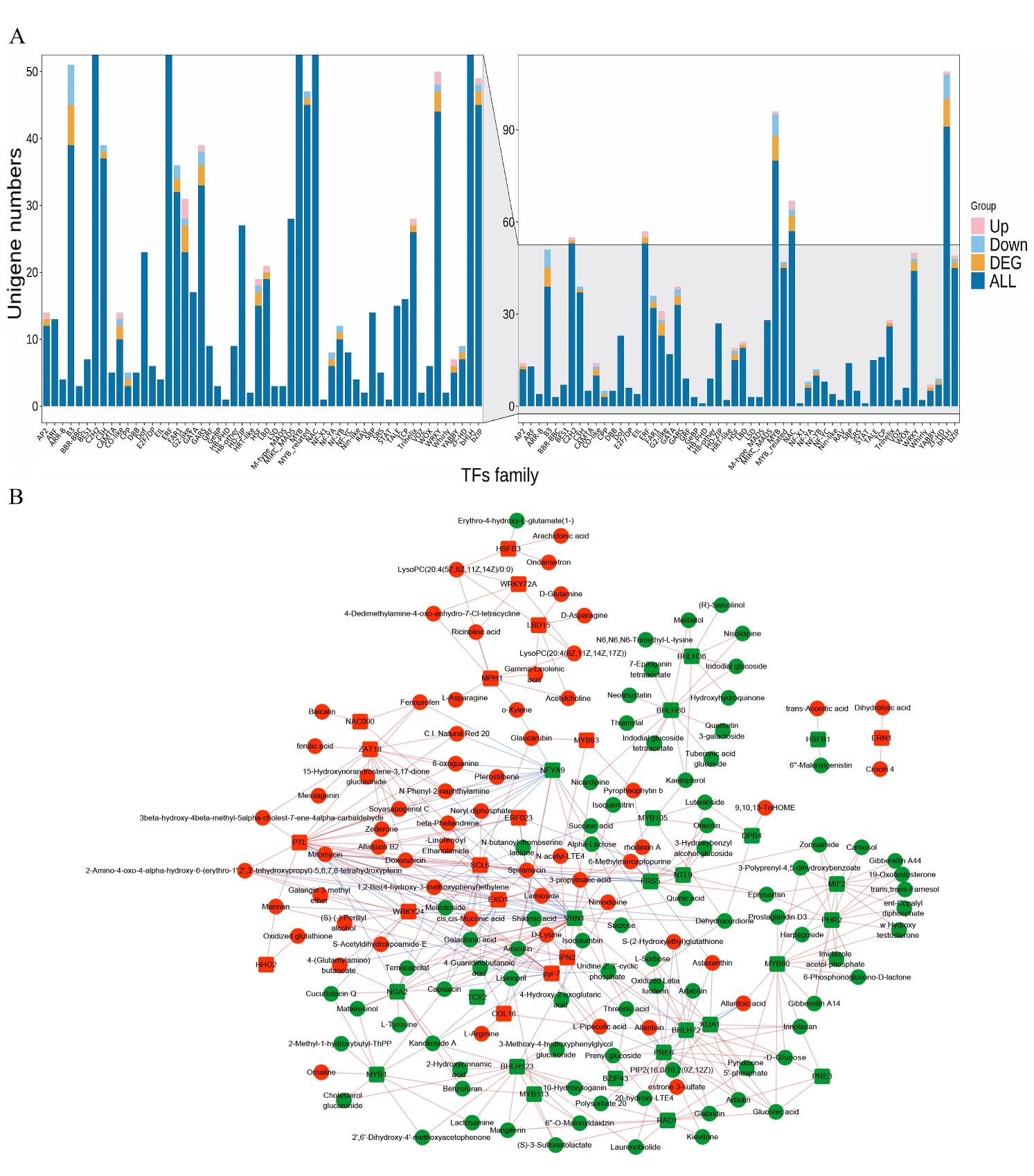


**Supplementary Figure 3.** Analysis of transcription factors (TFs) associated with polyploidization. **(A)** Classification of polyploidy-altered TFs. **(B)** Network of polyploidy-altered TFs and metabolites (|PCC |> 0.917). The red and green patterns represent upregulated and downregulated TFs or metabolites. DEG, differentially expressed genes.


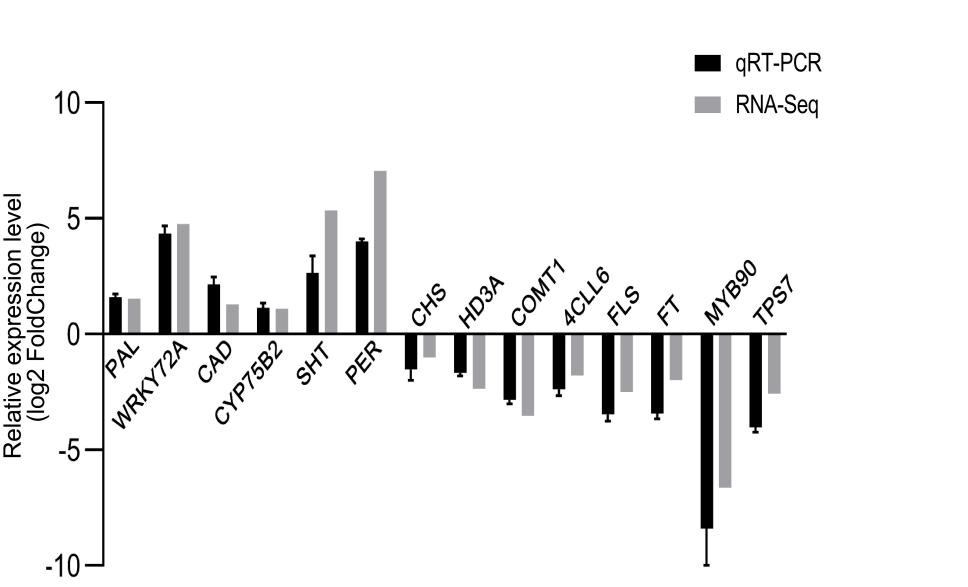


**Supplementary Figure 4.** Quantitative real-time polymerase chain reaction (qRT-PCR) validation of selected genes.


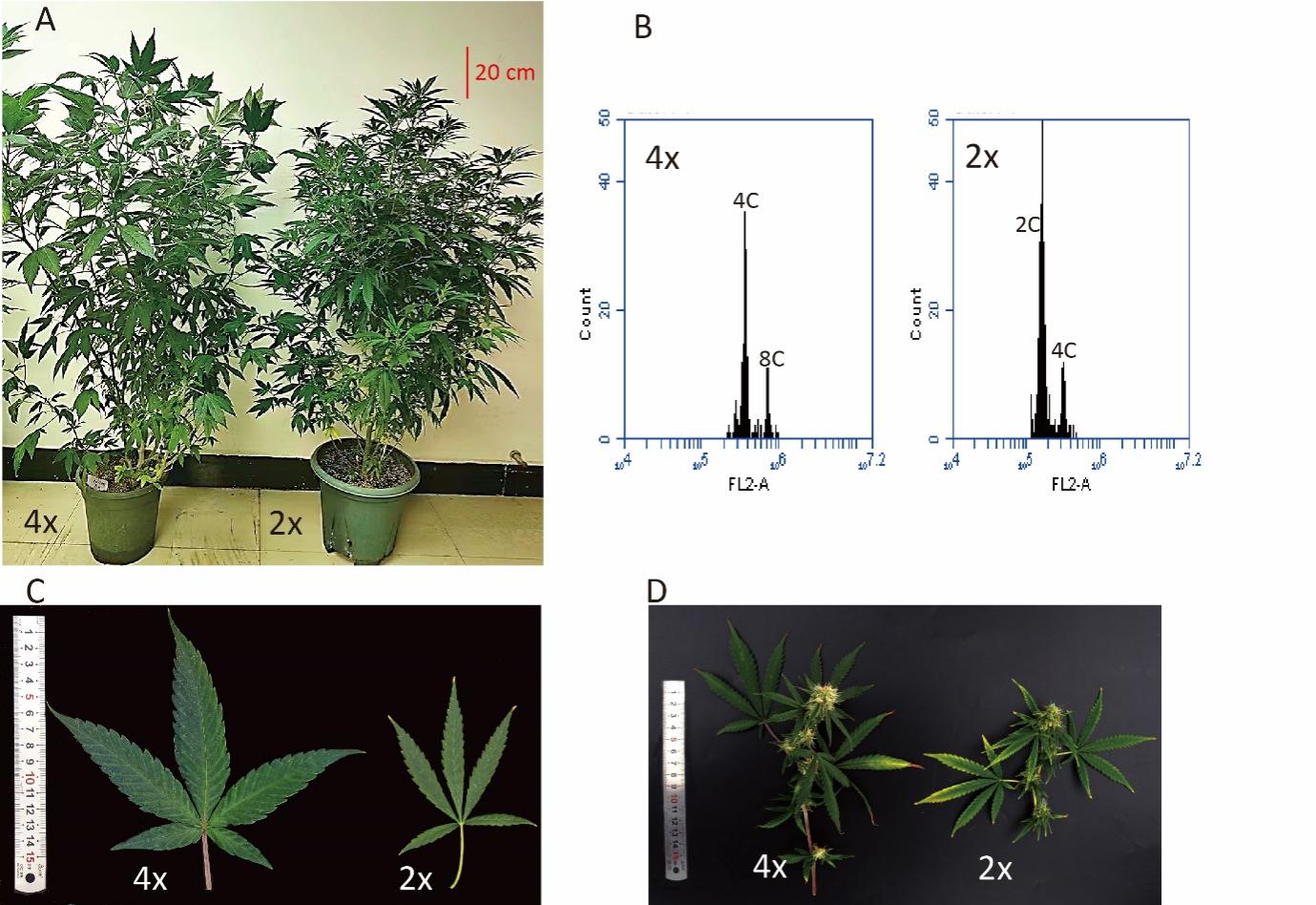


**Supplementary Figure 5.** Morphological characteristics of *Cannabis sativa* autotetraploid and its diploid progenitor. **(A)** The autotetraploid (4x) plant and its diploid (2x). **(B)** Result of flow cytometric analysis. **(C)** Comparison of leaf between autotetraploid and its diploid. **(D)** Comparison of flowers of autotetraploid and diploid plants.


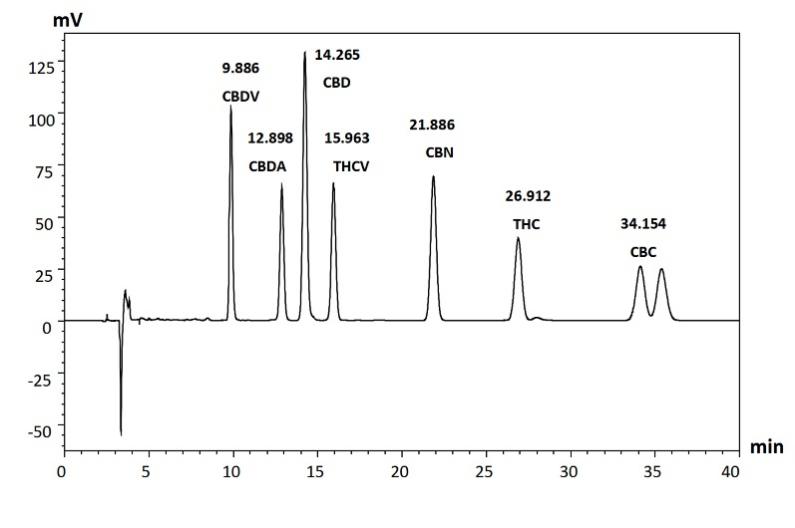


**Supplementary Figure 6.** The typical chromatogram of several cannabinoids.

**Supplementary Table 1.** Cannabinoids content (mean ± SE) for dried flower material of *Cannabis sativa* diploids and atutotetraploids analyzed in duplicated (n=9) by HPLC.

| Cannabinoid | Content (mg/g) | |
| --- | --- | --- |
|  | Cs2x | Cs4x |
| CBD | 66.3±2.2 | 66.2±0.1.3 |
| THC | 2.8±0.1 | 2.8±0.1 |
| CBDV | 4.5±0.1 | 3.5±0.3 |
| THCV | 1.7±0.1 | 1.1±0.1 |
| CBN | 0.8±0.1 | 0.8±0.2 |
| CBC | 0.6±0 | 0.7±0.1 |

**Supplementary Table 4.** DEGs related to phenylpropanoid biosynthesis.

| Gene id | Gene symbol | log2 Fold Change | Annotation |
| --- | --- | --- | --- |
| LOC115711262 | *PAL* | 1.526 | phenylalanine ammonia-lyase-like |
| LOC115703767 | *COMT1* | -3.536 | caffeic acid 3-O-methyltransferase-like |
| LOC115716906 | *COMT1* | 2.362 | caffeic acid 3-O-methyltransferase-like |
| LOC115708264 | *COMT1* | -2.484 | caffeic acid 3-O-methyltransferase-like |
| LOC115715671 | *4CLL6* | -1.7790 | 4-coumarate--CoA ligase-like 6 |
| LOC115702688 | *SALAT* | -7.851 | salutaridinol 7-O-acetyltransferase-like |
| LOC115703085 | *SALAT* | -9.210 | salutaridinol 7-O-acetyltransferase-like |
| LOC115704377 | *SALAT* | -6.8880 | salutaridinol 7-O-acetyltransferase-like |
| LOC115704444 | *SAT* | -2.521 | stemmadenine O-acetyltransferase-like |
| LOC115707958 | *HST* | -1.298 | shikimate O-hydroxycinnamoyltransferase-like |
| LOC115721427 | *HST* | 2.719 | shikimate O-hydroxycinnamoyltransferase-like |
| LOC115716544 | *SHT* | 5.3469 | spermidine hydroxycinnamoyl transferase-like |
| LOC115712971 | *FAOMT* | 1.074 | flavonoid 3',5'-methyltransferase-like isoform X2 |
| LOC115724170 | *CHS* | -1.007 | naringenin-chalcone synthase |
| LOC115702751 | *CAD* | 1.284 | probable cinnamyl alcohol dehydrogenase |
| LOC115709902 | *BGLU44* | -1.795 | beta-glucosidase 44-like isoform X1 |
| LOC115698821 |  | 1.655 | peroxidase P7-like |
| LOC115700070 | *PER16* | -1.015 | peroxidase 16-like |
| LOC115707710 | *PER29* | 1.183 | peroxidase 29-like |
| LOC115707258 | *PER10* | 3.057 | peroxidase 10-like |
| LOC115712307 |  | 5.191 | peroxidase 5-like |
| LOC115708062 |  | -4.673 | peroxidase 4-like |
| LOC115719602 |  | 1.113 | peroxidase 4-like |
| LOC115716054 |  | -3.382 | peroxidase 5-like |
| LOC115722136 |  | 1.678 | peroxidase 5-like |
| LOC115723064 |  | 7.046 | lignin-forming anionic peroxidase-like |
| LOC115717395 | *FLS* | -2.504 | flavonol synthase/flavanone 3-hydroxylase |
| LOC115709313 | *CYP75B2* | -1.435 | flavonoid 3'-monooxygenase |
| LOC115709845 | *CYP75B2* | 1.098 | flavonoid 3'-monooxygenase-like isoform X1 |
| LOC115716756 | *ANS* | -1.320 | leucoanthocyanidin dioxygenase-like |

**Supplementary Table 5.** DEGs related to monoterpenoid, and sesquiterpenoid and triterpenoid biosynthesis pathways.

| Gene id | log2 Fold Change | Gene symbol | Annotation | Pathway description |
| --- | --- | --- | --- | --- |
| LOC115704491 | -2.315 | *SDR1* | uncharacterized protein LOC115704491 | Monoterpenoid biosynthesis |
| LOC115706549 | -2.040 | *SDR1* | uncharacterized protein LOC115704491 | Monoterpenoid biosynthesis |
| LOC115695567 | -3.701 |  | (-)-germacrene D synthase-like | Sesquiterpenoid and triterpenoid biosynthesis |
| LOC115707439 | -2.581 | *TPS7* | (E,E)-alpha-farnesene synthase-like | Sesquiterpenoid and triterpenoid biosynthesis |
| LOC115714620 | -7.314 | *OSCBPY* | beta-amyrin synthase-like | Sesquiterpenoid and triterpenoid biosynthesis |
| LOC115719748 | -1.208 | *OSCBPY* | beta-amyrin synthase-like isoform X1 | Sesquiterpenoid and triterpenoid biosynthesis |
| LOC115719749 | -1.277 | *OSCBPY* | beta-amyrin synthase-like | Sesquiterpenoid and triterpenoid biosynthesis |

**Supplementary Table 9.** The primers of genes for qRT-PCR.

| **Gene id** | **Gene symbol** | **Primer name** | **Sequence (5' to 3')** |
| --- | --- | --- | --- |
| LOC115708959 | *MYB90* (*CsMYB82**) | q Cs 89592F | GCTCTGCACCAGTACCACAT |
|  |  | q Cs 8959 2R | TCCTCGCTGTCGATCATGTC |
| LOC115724170 | *CHS* | q Cs 4170 1F | GCCAGGCCCTATTTGGTGAT |
|  |  | q Cs 4170 1R | ATAGTTTGGGCCGCAGAGAC |
| LOC115711262 | *PAL* | q Cs 1262 1F | AGAGTCATGCCACACTCTGC |
|  |  | q Cs 1262 1R | TTGGCCATGGCTTCCAAGAT |
| LOC115715671 | *4CLL6* | q Cs 5671 1F | ATTGCTCCTGCGGATTTGGA |
|  |  | q Cs 5671 1R | GCCACTGGTATTTCCCCACA |
| LOC115703767 | *COMT1* | q Cs 3767 1F | AGATGTTGGGGGTGGTTTGG |
|  |  | q Cs 3767 1R | TGTCTCCACCCACATGTTCG |
| LOC115709845 | *CYP75B2* | q Cs 9845 2F | CCTTTCGTCTACACCCGTCC |
|  |  | q Cs 9845 2R | GGTCAGTCCATTGGTCTGGG |
| LOC115717395 | *FLS* | q Cs 7395 1F | CCCCAATGATGTCCCTGGTC |
|  |  | q Cs 7395 1R | CTGGGCCTGATCACCAATGT |
| LOC115716544 | *SHT* | q Cs 6544 2F | CGAGTCTCTCCCAATCCACG |
|  |  | q Cs 6544 2R | ACGCTCAGTCCATCAACGAG |
| LOC115723064 | *PER* | q Cs 3064 1F | CGGAGTTGTTTCTTGTGCCG |
|  |  | q Cs 3064 1R | AAGATCGCTCTCAGCTTCGG |
| LOC115702751 | *CAD* | q Cs 2751 2F | AATGAACACTACTGCGCCGA |
|  |  | q Cs 2751 2R | TGCTTACGCTTGGTGAACCA |
| LOC115707439 | *TPS7* | q Cs 7439 1F | GCCCGAAAGCATATCCGAGA |
|  |  | q Cs 7439 1R | ACTCGAGCAGTGTTAAGGGC |
| LOC115709155 | *WRKY72A* | q Cs 9155 1F | AAGATGTGCGGACGACATGT |
|  |  | q Cs 9155 1R | TTGTTGAACCGGAGAGGAGC |
| LOC115709185 | *FT* | q Cs 9185 1F | GCTCCTAACCCTAGCGAACC |
|  |  | q Cs 9185 1R | GAATTCCCACCGTCGGTCTT |
| LOC115696989 | *HD3A* | q Cs 6989 1F | TTTGGTCATGGTGGATCCGG |
|  |  | q Cs 6989 1R | CGTAGCACACCACCTCTTGT |
| LOC115725285 | *TUB* | q Cs TUB-F | CTCGGCTGAGAAAGCATACC |
|  |  | q Cs TUB-R | CCATGCCTAGGGTCACACTT |

* The name is according to the Bassolino et al., annotation. Reference: Bassolino L, Buti M, Fulvio F, Pennesi A, Mandolino G, Milc J, Francia E, Paris R. In silico identification of MYB and bHLH families reveals candidate transcription factors for secondary metabolic pathways in *Cannabis sativa* L. Plants. 2020;9(11):1540.
